# Suicidal ideation among the hypertensive individuals in Shandong, China: a path analysis

**DOI:** 10.1186/s12888-019-2256-7

**Published:** 2019-09-02

**Authors:** Dandan Ge, Xinyi Zhang, Xiaolei Guo, Jie Chu, Long Sun, Chengchao Zhou

**Affiliations:** 1 School of Public Health, Shandong University, Jinan, 250012 China; 2 Shandong Center for Disease Control and Prevention, Jinan, China; 3 Shandong Centre for Disease Control and Prevention, Jinan, 250014 China; 4 School of Public Health, NHC Key Lab of Health Economics and Policy Research, Shandong University, Jinan, 250012 China

**Keywords:** Hypertension, Suicidal ideation, Psychological distress, Path analysis

## Abstract

**Background:**

Although massive studies have explored the risk factors of suicidal ideation (SI), the prevalence of SI and its associated factors in the hypertensive individuals are largely unknown. This study aims to investigate the factors associated with SI in the hypertensive individuals.

**Methods:**

Three thousand nine hundred eleven hypertensive individuals in Shandong, China were included in the analysis. SI was assessed by using a question from the NCS (National Comorbidity Survey). We used binary logistic regression analysis to explore the factors associated with SI, and path analysis to test the direct and indirect relationships between associated factors and SI among hypertensive patients.

**Results:**

The prevalence of SI in the hypertensive individual was19.6%.

Psychological distress had the greatest direct (β = 0.640, *p*-value <0.01) and total effect (β = 0.640, *p*-value <0.01) on SI. Other factors including comorbidity (β = 0.090, *p*-value <0.01), gender (β = 0.088, *p*-value <0.01), marital status (β = − 0.037, *p*-value <0.01), economic status (β = − 0.106, *p*-value <0.01), residence (β = − 0.050, *p*-value <0.01), alcohol use (β = 0.011, *p*-value <0.01), exercise (β = − 0.114, *p*-value <0.01), hospitalization (β = 0.041, *p*-value <0.01) only had indirect effects on SI. Psychological distress was a mediator between SI and those variables.

**Conclusion:**

A significant mediation effect of psychological distress on the associations between SI and some associated factors (i.e., economic status, comorbidity) was demonstrated.

## Background

A global brief on hypertension by WHO reported that, the prevalence of hypertension in adults aged 25 and above was about 40% around the world in 2008, rising from 600 million in 1980 to 1 billion in 2008 during the past 28 years [1]. Similarly, this prevalence among the adults aged 18 and above in China has risen from 18.8% in 2002 to 25.2% in 2015, Presently, there are more than 200 million hypertensive patients in China [2]. Moreover, hypertension is a common cardiovascular and cerebrovascular disease which plays a major role in the development of cerebrovascular disease, cardiac and renal failure [3]. It was estimated that, 2.5 million Chinese adults died from hypertension and its comorbidities, accounting for 28% of all death in 2013 [4]. Hypertension is the leading risk factor for cardiovascular disease and premature death, which is an important public health problem in China [5, 6].

Suicidal ideation (SI) is a significant risk factor for suicidal attempt and suicide, which is a momentous part and inevitable stage of suicidal behavior [7, 8]. Many previous studies have shown that the SI was associated with chronic physical conditions (asthma, rheumatoid arthritis and hypertension) [9–11]. Some observed relationship between hypertension and mental health showed that hypertensive patients found to manifest symptoms of depression and stress, which were strongly related to SI [12]. In addition, a study based on DEPSCREEN-INFO clinical trial pointed out that hypertension was a clinical vulnerability of SI [11].

To the best of our knowledge, numerous studies have explored the risk factors of SI [11–13]. However, few studies have explored the SI and its associated factors in the hypertensive individuals, and no studies have examined the direct and indirect relationship between associated factors and SI among the hypertensive patients in China.

The current study aims to identify the risk factors of SI among the hypertensive patients. Firstly, this study will analyze the prevalence of the SI among the hypertensive patients. Secondly, this study will compare the SI across different subgroups of the hypertensive patients. Finally, using a path analysis method, this study will explore the direct and indirect relationship between associated factors and SI among the hypertensive patients in China.

## Methods

### Subjects

This study was conducted in Shandong Province, from August to October, 2016. Shandong ranks the second in the number of population in China. The prevalence of hypertension in the adults aged 18 to 69 in 2013 was 27.9%, which was significantly higher than the average level in the whole country [2]. A three-stage stratified cluster sampling method was used to select the participants. First, according to the proportion of urban to rural residents (1 to 2) and Gross Domestic Product (GDP) per capita in Shandong in 2015, we selected two urban districts (one upper the medium GDP level, Fushan, and the other below the medium GDP level, Weicheng) and four rural counties (one upper the medium level, Rushan, two at the medium level, Yiyuan, and Gaotang, and one below the medium level, Liangshan) as study sites. Second, according to GDP per capita, all of the sub-districts and townships in each selected urban districts and rural counties were divided into three levels. For each level, one sub-district and one township were randomly selected. Third, we randomly selected two communities or villages with more than 1000 permanent residents from each of the selected sub-districts and townships. More details about data collection methods were reported in our previous studies [14].

The criterion for hypertension was systolic blood pressure (SBP) ≥ 140 mm HG (1 mm HG = 0.133 k Pa) and diastolic blood pressure (DBP) ≥ 90 mm HG, following the Chinese guideline for the management of hypertension in 2005 [15]. In addition, the standards for hypertension diagnosis have been further strengthened since 2010 [16]. In this study, all hypertensive patients who have already been diagnosed as hypertension by medical professionals of different-level medical institutions and registered in the sample community or village chronic case management system were included. We excluded those who were unable to communicate with the interviewers properly. In total, 3911 hypertensive patients were included in the current study.

### Data collection

The healthcare practitioners in sample community health stations or village clinics were responsible for recruiting the hypertensive patients in the list of chronic case management system. About 1 week before the normal survey, the hypertensive patients were informed of the purpose and time of the interview by the healthcare practitioners and then signed the willingness for the participation in the survey.

All the subjects were interviewed face-to-face using a standard structured questionnaire by trained postgraduate students from Shandong University School of Public Health. Before the interview, the written consent were obtained from each of the participants. To achieve the purposes of this study, completed questionnaires were subject to stringent quality assurance.

### Variables

#### Social demographic characteristics

The social demographic characteristics of the participants included age, gender, education, marital status, residence, and economic status. When measuring “Economic status”, we used a question of “How much was the total income of your household in the past year (Urban households were disposable income, rural households were net income)?” It was divided into four same group distance: 25% (Quartile 1,Q1), 50% (Quartile 2,Q2),75% (Quartile 3,Q3), and 100% (Quartile 4,Q4), and Q1 was the poorest, and Q4 was the richest.

#### Health behaviors

The behavioral variables were assessed using smoking, alcohol use, and exercise. These characteristics were defined as follows: smoking (current smokers and the cumulative quantity is more than100 cigarettes before the survey, yes vs. no) [17–19], alcohol use (drinking on any day in the past 30 days, yes vs. no) [20, 21], exercise (< 1, 1–2, > = 3 times a week).

#### Variables associated with hypertensive health

Variables associated with hypertensive health were obtained through types of anti-hypertension drugs in the previous 2 weeks (0, 1, ≥2), hospitalization due to hypertension in the past year (yes vs. no), and duration of hypertension (< 5, 5-, 15-, 20+ years).

#### Psychological distress (K10)

This study applied 10-item Kessler Psychological Distress Scale which was composed of 10 items to evaluate the status of psychology distress, mainly focusing on depression and anxiety during the past 30 days. This scale was used widely in various countries for screening psychological distress [22, 23]. Each item includes five dimensions from “none of the time” to “all of the time”, and is scored from 1 to 5. A total score of 10 to19 was coded into1, which was suggestive of lower risk of suffering from mental illness; 20 to 24 points was coded into 2, which was indicative of low risk of a mental illness; 25 to 29 points was coded into 3, which was suggestive of relatively high risk of psychiatric disorders, and 30 to 50 was coded into 4, which was indicative of higher risk of mental disorders [24]. The Cronbach’s α of the K10 in this study was 0.89.

#### Suicidal ideation

A question of “Have you ever seriously considered committing suicide?” was used to measure SI in this study. If the answer was ‘yes’, SI was coded as ‘1’, and if the answer was ‘no’, SI was coded as ‘0’. The question comes from the baseline NCS (National Comorbidity Survey), which has been widely used in many previous studies with high reliability and validity [25, 26].

#### Statistical analysis

All data were analyzed by using Mplus7.0 and SPSS 22.0.We used percentages to describe the demographics and Chi-square test to compare the prevalence of SI across different subgroups of the hypertensive patients. Binary logistic regression models were employed to explore the factors associated with SI. We also used a path analysis model to identify the direct and indirect effect of associated factors on SI. The statistical significance was evaluated at the level of 5%. In order to avoid potential alpha inflation, we adjusted the significance level by the number of multiple tests in the multiple models.

## Results

Of the 3911 subjects, 767 (19.6%) had SI. The patients with comorbidities had a significantly higher prevalence of SI (25.2%) compared with those without comorbidities (16.8%). The prevalence of SI among singles was 27.0%, which was higher than those with a couple (18.0%). The hypertensive individuals from rural areas had a higher prevalence of SI (21.6%) than those from urban areas (12.0%). The current alcohol users had a higher SI rate (21.3%) than their counterparts (11.3%). Those patients who had hospitalization had a significantly higher prevalence of SI (26.9%) than those without hospitalization due to hypertension in the previous year (18.5%). As for the psychological well-being, the patients with higher level of K10 were more likely to experience SI (See the Table 1).

**Table 1:** Factors associated with suicidal ideation among the hypertensive patients in Shandong, China, 2016

Characteristics	Total n (%)	Suicidal Ideation		*P*
		Yes (%)	No (%)	
Observation	3911	767 (19.6)	3144 (80.4)	
Comorbidity				**0.000**
Yes	1326 (33.9)	334 (25.2)	992 (74.8)	
No	2585 (66.1)	433 (16.8)	2152 (83.2)	
Gender				**0.000**
Male	1433 (36.6)	202 (14.1)	1231 (85.9)	
Female	2478 (63.4)	565 (22.8)	1913 (77.2)	
Age				0.277
<60	847 (21.7)	155 (18.3)	692 (81.7)	
≥60	3064 (78.3)	612 (20.0)	2452 (80.0)	
Marital status				**0.000**
Single ^a^	686 (17.5)	185 (27.0)	501 (73.0)	
Married	3225 (82.5)	582 (18.0)	2643 (82.0)	
Education				
Illiteracy or semiliterate	1502 (38.4)	349 (23.2)	1153 (76.8)	**0.000**
Primary school	1363 (34.9)	272 (20.0)	1091 (80.0)	
Junior school	750 (19.2)	112 (14.9)	638 (85.1)	
Senior school or above	296 (7.6)	34 (11.5)	262 (88.5)	
Economic status ^b^				**0.000**
Q1	982 (25.1)	250 (25.5)	732 (74.5)	
Q2	976 (25.0)	220 (22.5)	756 (77.5)	
Q3	977 (25.0)	187 (19.1)	790 (80.9)	
Q4	976 (25.0)	110 (11.3)	866 (88.7)	
Residence				**0.000**
Rural	3113 (79.6)	671 (21.6)	2442 (78.4)	
Urban	798 (20.4)	96 (12.0)	702 (88.0)	
Smoking				**0.000**
No	2987 (76.4)	647 (21.7)	2340 (78.3)	
Yes	924 (23.6)	120 (13.0)	804 (87.0)	
Alcohol use				**0.000**
No	3256 (83.3)	693 (21.3)	2563 (78.7)	
Yes	655 (16.7)	74 (11.3)	581 (88.7)	
Exercise (time/a week)				**0.000**
<1	1588 (40.6)	393 (24.7)	1195 (75.3)	
1–2	214 (5.5)	43 (20.1)	171 (79.9)	
≥3	2109 (53.9)	331 (15.7)	1778 (84.3)	
Number of medicine				0.718
0	455 (11.6)	83 (18.2)	372 (81.8)	
1	2300 (58.9)	465 (20.2)	1835 (79.8)	
≥2	1156 (29.5)	219 (19.0)	937 (81.0)	
Duration				0.202
<5	1230 (31.4)	221 (18.0)	1009 (82.0)	
5-	1234 (31.6)	239 (19.4)	995 (80.6)	
15-	531 (13.6)	115 (21.7)	416 (78.3)	
20+	916 (23.4)	192 (21.0)	724 (79.0)	
Hospitalization				**0.000**
No	3371 (86.2)	622 (18.5)	2749 (81.5)	
Yes	540 (13.8)	145 (26.9)	395 (73.1)	
K10^c^				**0.000**
1	2358 (60.3)	144 (6.1)	2214 (93.9)	
2	624 (16.0)	164 (26.3)	460 (73.7)	
3	499 (12.8)	205 (41.1)	294 (58.9)	
4	430 (11.0)	254 (59.1)	176 (40.9)	

Note: *P-values* indicate statistical significance at 5% level

^a^Singles include those who are unmarried (1.7%), divorced (0.3%), widowed (15.5%)

^b^Quartile 1(Q1) is the poorest and Quartile 4(Q4) is the richest

^c^K10 means 10-item Kessler Psychological Distress Scale:1 was suggestive of lower risk of suffering from mental illness, 2 was indicative of low risk of a mental illness,3 was suggestive of relatively high risk of psychiatric disorders, and 4 was indicative of higher risk of mental disorders

We presented the results of this study to explore the relationships between related variables and SI using two models. In model 1, we included variables except K10. The model showed that the variables including comorbidity, gender, marital status, economic status, residence, alcohol use, exercise, and hospitalization were significantly associated with SI. When we included K10 in Model 2, economic status and marital status were still significantly associated with SI (See the Table 2).

**Table 2:** Multivariate logistic regression models for factors associated with suicidal ideation among the hypertensive patients in Shandong, China, 2016

Characteristics	Model 1			Model 2				
	OR	95% CI		*P*-value	OR	95% CI		*P*-value
Comorbidity								
No	1.00				1.00			
Yes	1.41	1.18	1.69	**0.000**	1.06	0.87	1.30	0.542
Gender								
Male	1.00				1.00			
Female	1.32	1.03	1.69	**0.028**	1.03	0.79	1.36	0.820
Marital status								
Single^a^	1.00				1.00			
Married	0.70	0.57	0.87	**0.001**	0.79	0.62	0.99	**0.043**
Education								
Illiteracy or semiliterate	1.00				1.00			
Primary school	1.09	0.90	1.32	0.374	1.19	0.96	1.47	0.113
Junior school	0.95	0.73	1.22	0.672	1.05	0.79	1.39	0.737
Senior school or above	0.85	0.57	1.28	0.442	0.89	0.57	1.40	0.625
Economic status ^b^								
Q4	1.00				1.00			
Q1	1.85	1.39	2.46	**0.000**	1.23	0.90	1.68	0.199
Q2	1.81	1.37	2.39	**0.000**	1.32	0.98	1.79	0.069
Q3	1.56	1.19	2.06	**0.001**	1.35	1.00	1.82	**0.047**
Residence								
Urban	1.00				1.00			
Rural	1.37	1.05	1.80	**0.021**	1.25	0.93	1.68	0.134
Smoking								
No	1.00				1.00			
Yes	0.78	0.58	1.03	0.079	0.74	0.54	1.01	0.055
Alcohol use								
No	1.00				1.00			
Yes	0.72	0.53	0.97	**0.030**	0.91	0.66	1.26	0.567
Exercise (time/a week)								
<1	1.00				1.00			
1–2	0.74	0.52	1.06	0.096	1.17	0.44	1.26	0.439
≥3	0.57	0.48	0.67	**0.000**	0.88	0.73	1.07	0.198
Hospitalization								
No	1.00				1.00			
Yes	1.38	1.09	1.74	**0.007**	1.18	0.91	1.53	0.218
K10^c^								
1					1.00			
2					4.95	3.85	6.36	**0.000**
3					9.34	7.25	12.03	**0.000**
4					18.94	14.46	24.81	**0.000**

Note: The *P-values* indicate statistical significance at 5% level

^a^Singles include those who are unmarried (1.7%), divorced (0.3%), widowed (15.5%)

^b^Quartile 1(Q1) is the poorest and Quartile 4(Q4) is the richest

^c^K10 means 10-item Kessler Psychological Distress Scale

**Fig. 1:**
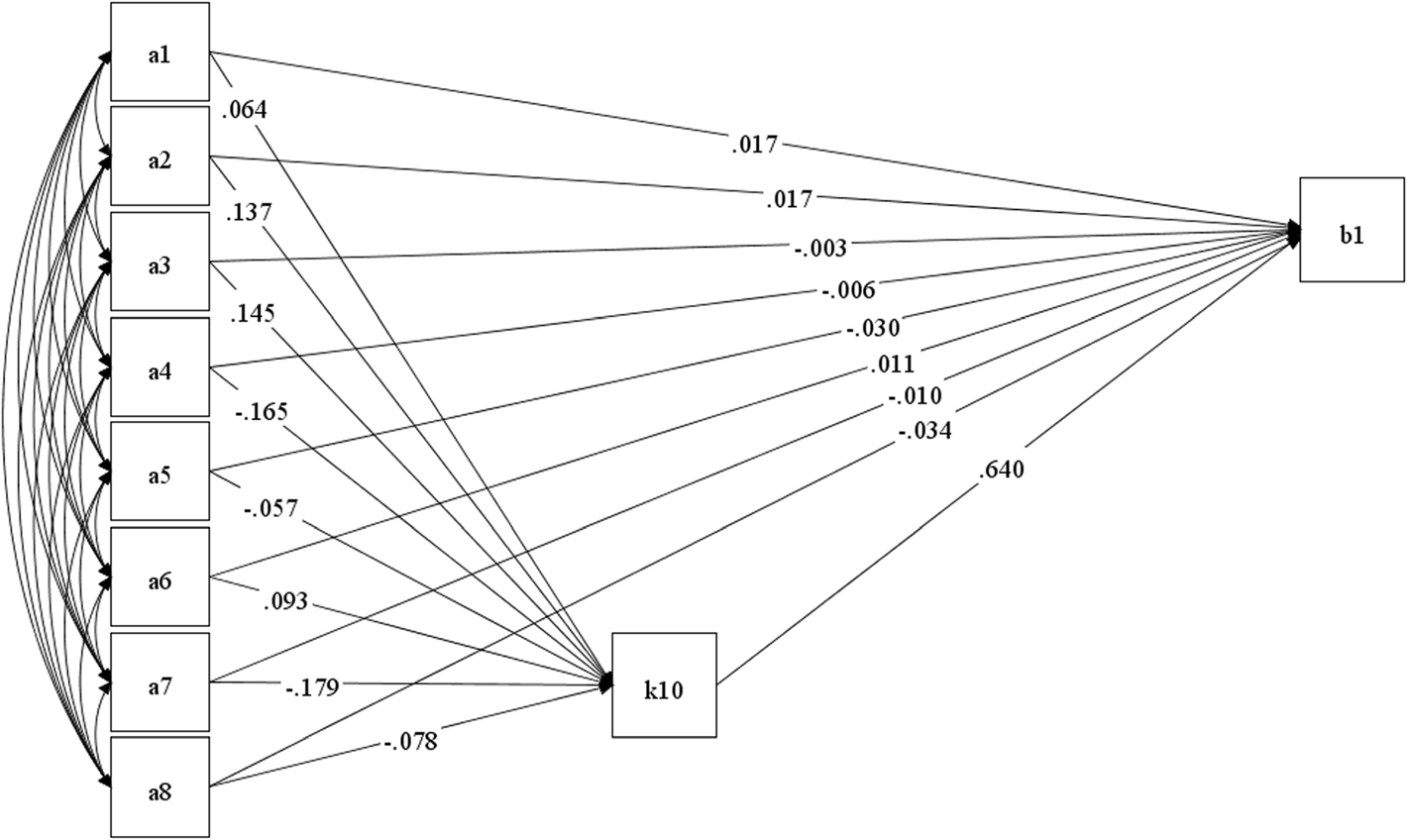
Path analysis of suicidal ideation among the hypertensive individuals in Shandong (*n* = 3911), China, 2016. k10: Psychological distress, a1: Hospitalization, a2: Gender, a3: Comorbidity, a4: Economic status, a5: Marital status, a6: Alcohol use, a7: Exercise, a8: Residence, b1: Suicidal ideation

We used a path analysis model to further explore the relationship between SI and related variables in hypertensive patients. As shown in Table 3 and Fig. 1, nine specified factors (comorbidity, gender, marital status, economic status, residence, alcohol use, exercise, hospitalization and K10) included in the model. Standardized total effects of the greatest absolute value was K10 (0.640), followed by exercise (− 0.124), economic status (− 0.112). We found that K10 had both direct impact on SI, and the other eight variables was mediated by K10 towards SI. Goodness of fit indices was employed to measure the model fit in this study. In this model, TLI (tucker-lewis index) had a value of 1.00, CFI (comparative fit index) value 0.99, and a root mean square error of approximation (RMSEA) value 0.000. The overall variables accounted for 44.1% of the variance in SI. All of these indexes have confirmed the path model.

**Table 3:** Standardized effects on suicidal ideation from path analysis among the hypertensive patients in Shandong, China, 2016

	Total effect	Direct effect	Indirect effect
Gender	0.105^**^	0.017	0.088^**^
Marital status	−0.066^*^	−0.030	−0.037^**^
Economic status	−0.112^**^	−0.006	−0.106^**^
Residence	−0.084^*^	−0.034	−0.050^**^
Alcohol use	0.071^*^	0.061	0.011^**^
Exercise	−0.124^**^	−0.010	−0.114^**^
Hospitalization	0.058^*^	0.017	0.041^**^
Comorbidity	0.090^**^	−0.003	0.090^**^
K10^a^	0.640^**^	0.640^**^	–

Note: The total effects are the sum of the direct and indirect effects;

^*^: *P* < 0.05, ^**^: *P* < 0.01

^a^K10 means 10-item Kessler Psychological Distress Scale

## Discussion

Our study provided for the first time a real profile of SI among the hypertensive individuals in China. In this study, we found that the prevalence of lifetime SI among the hypertensive was 19.6%. It was higher than the reported incidence of SI (13.5%) in adults aged from 15 to 54 by Kessler et al. in 1999 [27]. It was also much higher than the reported SI rate in rural and urban residents in Beijing (2.3%), older adults aged 60 and above (4.2%), community-dwelling elders in Taiwan (6.1%), and medical college students (12.9%) in China [28–31]. When compared with those with chronic diseases, we also found that it was higher than the reported SI rate of 8.7% in the individuals with asthma and 12.0% in the patients with COPD (chronic obstructive pulmonary disease) in United States [32, 33]. This was also higher than the reported SI rate of 11% in the hospital outpatient with rheumatoid arthritis in United Kingdom and Chinese patients with COPD (15.4%) [10, 34]. The prevalence of SI in the current study was lower than what was reported in patients with asthma (21.4%) in Korea, patients with AIDS during their lifetime (27.2%) in Changsha city, and also patients with ovarian cancer (30.3%) in Hunan Province, China [9, 35, 36]. Even though the SI rate in the current study is a little lower than those in the patients with AIDS or ovarian cancer, it is higher than those in general population and those patients with some other chronic diseases, which indicates that hypertension might be a serious type of chronic diseases that increase the risk of SI.

In this study, we found the psychological distress had the most potent effect (0.640) and direct impact (0.640) on SI. This finding was similar to the previous studies which indicated that psychological distress was the primary determinant of SI [37, 38]. Many studies have demonstrated that those who with high-level psychological distress would be more likely to have SI and suicidal plan [39–41]. Some studies showed that hypertension and psychological distress act together in a vicious cycle, and can sometimes lead to SI [42, 43]. Given the relationship between hypertension and psychological distress, we should develop interventions to decrease the prevalence of SI [44].

This study demonstrated that both hospitalization and comorbidity had indirect effect on SI mediated by psychological distress. Some studies revealed that hypertensive individuals with hospital admission during the previous year, or with comorbidities tend to experience mental illness and SI [45, 46]. A study in elderly patients with hypertension showed a high level of depression, and comorbidity is the primary influencing factor for depression [47]. Another study about the causes of SI among the elderly indicated that the seniors suffering from disability due to long-term chronic diseases had a significant impact on psychological disorders and SI [48]. Placido et al. pointed out that the incidence of suicide was positively correlated with those with ischemic heart disease and stroke [49]. This finding indicated that psychological distress played an important meditating role in the association between hospitalization, comorbidity and SI among the hypertensive patients.

Negative direct and indirect relationships were detected between economic status and SI, which were similar to previous studies. Some studies have demonstrated that financial situation was an associated factor for SI mediated by mental illness [50, 51]. Some studies also found people with financial difficulties were more likely to experience psychological disorders and suicide [52–54]. Similar to the previous studies, we also found marital status had an indirect effect on SI. Some studies demonstrated that the rate of SI in married patients was lower than that in as table married (unmarried, divorced, widowed) patients [55]. This might be due to the protection of social support from the family. Zhang et al. found that social support from family moderated the suicidal thoughts [56]. A study by Yang and Clum also found that mental illness was a mediator between social support and SI [57]. In this study, we also found gender had an indirect association with SI. A survey conducted by Feng et al. demonstrated that the female had a higher risk for SI than the male, which might be due to Chinese Confucianism [58]. In addition, our study showed that healthy lifestyles, including exercise and non-use of alcohol, had an adverse effect on SI, which was consistent with some previous studies [59, 60].

Interestingly, it was observed that all variables related to SI were mediated by psychological distress. The current study demonstrated a close and significant relationship between psychological distress and SI in hypertensive patients. A study conducted by Bruce et al. in the USA indicated that depression was the primary risk factor of SI, and an intervention of primary care can efficiently reduce the prevalence of SI in the depressive patients [61]. It was vital for the policymakers to develop interventions to regularly monitor the psychological well-being in the hypertensive individuals for early detection of psychological disorders, especially for those at-risk subgroups (e.g., those hypertensive patients with comorbidities, hospitalization use or poor socioeconomic status), so as to prevent the potential SI in the hypertensive individuals in China.

The current study is restricted to the hypertensive patients, the associations between identified factors and SI, and also the path model, might be somewhat specific for the hypertensive patients. In addition, the coefficients of the path analysis were high. Hence, future work will focus on examining whether the associations and path model can be generalized to other populations.

This study had some limitations. First, as a cross-sectional survey, the associations between identified factors and SI measured in this study could not be interpreted as causality. In addition, owing to the limitation of the cross-sectional study, we could only measure the prevalence of SI, but could not assess the incidence of the SI. Second, in this study, we just concentrated on the associated factors for SI, and gave no concerns about some other suicidal behaviors (e.g., plans, attempts), which would be remedied in the follow-up study. Third, the measurement of the SI mainly relied on one question and self-report information, which might result in inevitable bias. Fourth, the measurement of current smoking behavior was somewhat crude, and could not indicate the quantity, which would also be remedied in the follow-up study. Finally, some other possible confounding factors were not included in the analysis, which will be made up in the follow-up study.

## Conclusions

Psychological distress had the strongest direct and total effect on SI. Factors including comorbidity, gender, marital status, economic status, residence, alcohol use, exercise, and hospitalization only had indirect effects on SI. A significant mediation effect of psychological distress on the associations between SI and aforementioned risk factors (i.e., comorbidity, gender) was demonstrated. These findings of the path analysis would improve our understanding of the mechanisms of the effect of different associated factors on SI in hypertensive individuals and facilitate selection of intervention strategies by targeting at the associated factors, so as to prevent subsequent suicidal behaviors.

## Data Availability

Please contact the corresponding author for data requests.

## Abbreviations

CFI: comparative fit index
COPD: chronic obstructive pulmonary disease
DBP: diastolic blood pressure
GDP: Gross Domestic Product
K10: 10-item Kessler Psychological Distress Scale
NCS: National Comorbidity Survey
RMSEA: root mean square error of approximation
SBP: systolic blood pressure
SI: Suicidal ideation
TLI: tucker-lewis index
WHO: World Health Organization
